# A Manual of the Practice of Medicine

**Published:** 1896-06

**Authors:** 


A Manual of the Practice of Medicine. By George Roe Lockwood, M. D., Professor of Practice in the Woman's Medical College of the New York Infirmary; Attending Physician to the Colored Hospital and to the City (late Charity) Hospital, etc., etc. Small 8vo, pp. 935. With seventy-five illustrations in the text and twenty-two full-page colored plates. Price, $2.50. Philadelphia: W. B. Saunders, 925 Walnut street. 1895.

As the author says in his preface, he "aims at presenting the essential facts and principles of the practice of medicine in a concise and available form." The book is of convenient size, good type and the excellent classification of Osler has been adopted. There are many plates of typical temperature, charts and some fine diagrams in the chapters devoted to diseases of the heart, lungs and brain and many other illustrations besides. The style is easy and the writer comes to the point quickly by a process of reasoning so concise and simple that the overworked student and busy practitioner owe him gratitude. He is of a practical turn of mind and gives the reader the benefit of his own large and valuable experience. Diagnosis is fully dwelt upon; only enough pathology, however, is given to enable one to follow the rationality of the treatment. Dosages and prescriptions do not figure so largely as in many of our text-books on practice, but the indications for certain classes of drugs, modes of dieting, and the like, are clearly given, and individual judgment and individual patients may regulate the minutiæ. Brand's treatment of typhoid and the use of antitoxin in diphtheria are explained and the book is up to date in recommending the tested reliable therapeutic agents of recent note. The chapters devoted to diseases of the circulatory system are especially fine, logical and enjoyable, and the work as a whole is a valuable addition to the medical library. E. C.

